# Distinct Cerebrovascular Reactivity Patterns for Brain Radiation Necrosis

**DOI:** 10.3390/cancers13081840

**Published:** 2021-04-13

**Authors:** Giovanni Muscas, Christiaan Hendrik Bas van Niftrik, Martina Sebök, Alessandro Della Puppa, Katharina Seystahl, Nicolaus Andratschke, Michelle Brown, Michael Weller, Luca Regli, Marco Piccirelli, Jorn Fierstra

**Affiliations:** 1 Department of Neurosurgery, Department of Neuroscience, Psychology, Pharmacology and Child Health (NEUROFARBA) Careggi University Hospital, 50139 Florence, Italy; muscasgi@aou-careggi.toscana.it (G.M.); alessandro.dellapuppa@unifi.it (A.D.P.); 2 Department of Neurosurgery, University Hospital Zurich, University of Zurich, 8091 Zurich, Switzerland; bas.vanniftrik@usz.ch (C.H.B.v.N.); martina.seboek@usz.ch (M.S.); luca.regli@usz.ch (L.R.); 3 Clinical Neuroscience Center, University Hospital Zurich, 8091 Zurich, Switzerland; katharina.seystahl@usz.ch (K.S.); michael.weller@usz.ch (M.W.); marco.piccirelli@usz.ch (M.P.); 4 Department of Neurology, University Hospital Zurich, University of Zurich, 8091 Zurich, Switzerland; 5 Department of Radiation Oncology, University Hospital Zurich, University of Zurich, 8091 Zurich, Switzerland; nicolaus.andratschke@usz.ch (N.A.); michelleleanne.brown@usz.ch (M.B.); 6 Department of Neuroradiology, University Hospital Zurich, University of Zurich, 8091 Zurich, Switzerland

**Keywords:** cerebrovascular reactivity, MRI, glioma, radiation necrosis

## Abstract

**Simple Summary:**

Discrimination between radiation necrosis versus recurrent glioblastoma contrast-enhancing lesions remains imprecise but is paramount for prognostic and therapeutic evaluation. We examined whether patients with radiation necrosis exhibit distinct patterns of blood oxygenation-level dependent functional magnetic resonance imaging (fMRI) cerebrovascular reactivity (BOLD-CVR) and studied eight patients with primary and secondary brain tumors and a multidisciplinary clinical and radiological diagnosis of radiation necrosis and fourteen patients with a first diagnosis of glioblastoma. The contrast-enhancing lesion was derived from high-resolution T1-weighted MRI and rendered the volume-of-interest (VOI). From this primary VOI, an additional 3 mm concentric expanding VOIs up to 30 mm were created for a detailed perilesional BOLD-CVR. Mean intralesional BOLD-CVR values were markedly lower in radiation necrosis. Perilesionally, a characteristic BOLD-CVR pattern was observed for radiation necrosis. In this preliminary analysis, distinctive intralesional and perilesional BOLD-cerebrovascular re-activity patterns are found for radiation necrosis.

**Abstract:**

*Background:* Current imaging-based discrimination between radiation necrosis versus recurrent glioblastoma contrast-enhancing lesions remains imprecise but is paramount for prognostic and therapeutic evaluation. We examined whether patients with radiation necrosis exhibit distinct patterns of blood oxygenation-level dependent fMRI cerebrovascular reactivity (BOLD-CVR) as the first step to better distinguishing patients with radiation necrosis from recurrent glioblastoma compared with patients with newly diagnosed glioblastoma before surgery and radiotherapy. *Methods:* Eight consecutive patients with primary and secondary brain tumors and a multidisciplinary clinical and radiological diagnosis of radiation necrosis, and fourteen patients with a first diagnosis of glioblastoma underwent BOLD-CVR mapping. For all these patients, the contrast-enhancing lesion was derived from high-resolution T1-weighted MRI and rendered the volume-of-interest (VOI). From this primary VOI, additional 3 mm concentric expanding VOIs up to 30 mm were created for a detailed perilesional BOLD-CVR tissue analysis between the two groups. Receiver operating characteristic curves assessed the discriminative properties of BOLD-CVR for both groups. *Results:* Mean intralesional BOLD-CVR values were markedly lower in radiation necrosis than in glioblastoma contrast-enhancing lesions (0.001 ± 0.06 vs. 0.057 ± 0.05; *p* = 0.04). Perilesionally, a characteristic BOLD-CVR pattern was observed for radiation necrosis and glioblastoma patients, with an improvement of BOLD-CVR values in the radiation necrosis group and persisting lower perilesional BOLD-CVR values in glioblastoma patients. The ROC analysis discriminated against both groups when these two parameters were analyzed together (area under the curve: 0.85, 95% CI: 0.65–1.00). *Conclusions*: In this preliminary analysis, distinctive intralesional and perilesional BOLD-cerebrovascular reactivity patterns are found for radiation necrosis.

## 1. Introduction

In patients undergoing post-surgical radiotherapy for glioblastoma, the appearance of new contrast-enhancing lesions on clinical follow-up imaging poses a significant diagnostic challenge, as radiation necrosis and recurrent glioblastoma share similar radiological features on contrast-enhanced T1-weighted magnetic resonance imaging (MRI) [1,2,3,4].

However, reliable discrimination between radiotherapy-induced tissue changes versus tumor recurrence is paramount for prognostic purposes and therapeutic decision-making.

Advanced neuroimaging techniques, such as MR perfusion imaging (MRP), MR spectroscopy, and ^18^F-(fluoroethyl)-l-tyrosine positron emission tomography (FET-PET) [5,6,7], may have better discriminatory properties than morphological MRI but need further development to enhance diagnostic reliability [3,8,9].

In this regard, the application of blood oxygenation level-dependent fMRI cerebrovascular reactivity (BOLD-CVR) deserves consideration as an adjunct parameter. By using a standardized carbon dioxide challenge, quantitative whole-brain BOLD-CVR mapping with high imaging resolution can be achieved [10,11,12]. In previous studies, our group, as well as others, have shown that newly diagnosed glioblastoma is associated with impaired intra- and perilesional BOLD-CVR [12,13,14,15] and that these BOLD-CVR patterns may have merit in identifying brain areas at high risk for glioblastoma recurrence [16]. In this case, the impaired intra- and perilesional BOLD-CVR may be related to vascular dysregulation due to tumor neoangiogenesis and altered autoregulation due to the co-option of tumor cells accumulating around existing vasculature [14,17]. Therefore, the erratic growth behavior of glioblastoma lesions may also explain the prevalent perilesional BOLD-CVR impairment [12,13,18].

Since radiation necrosis is believed to have different biological properties by representing a confined inflammatory tissue response, specific intra- and perilesional BOLD-CVR patterns may be expected. Therefore, we examined whether patients with radiation necrosis exhibit characteristic BOLD-CVR patterns.

## 2. Materials and Methods

### 2.1. Patient Selection

This study was carried out under research protocol KEK-ZH-Nr. 2012-0427, and all participants signed informed consent before study participation. Radiation necrosis patients were consecutively screened and enrolled at the Clinical Neuroscience Center of the University Hospital Zurich from January 2017 to January 2018, using the following criteria: Newly detected contrast-enhancing lesion on contrast-enhanced T1-weighted MRI in the absence of new symptoms after radiotherapy, the lesion remained stable or regressed during at least six months post-radiation follow-up [2], with subsequent imaging (either FET-PET, MR spectroscopy (MRS), or MR perfusion (MRP)) findings supporting a multidisciplinary neuro-oncological board consensus of a radiation necrosis diagnosis. These patients did not receive steroids or bevacizumab therapy prior to the BOLD-CVR scan. Patients with suspected radiation necrosis showing lesion enlargement at follow-up were excluded. A reference cohort consisted of patients with newly diagnosed glioblastomas (WHO grade IV), who underwent an identical BOLD-CVR examination before microsurgical tumor resection. Histopathological diagnosis confirmation was available for all glioblastoma patients after surgery.

### 2.2. BOLD-CVR Acquisition and Calculation

BOLD-CVR examinations and calculations were done in accordance with our previously published protocol [19,20]. In particular, the novelty of this quantitative BOLD-CVR acquisition is related to the standardized application of the vasoactive stimulus [21]. Here, BOLD signal changes are induced by a standardized single hypercapnia pseudo-square wave that is generated from an iso-oxic carbon dioxide (CO_2_) stimulus applied by a custom-built computer-controlled gas blender (RepirAct^TM^, Thornhill Research Institute, Toronto, ON, Canada) [19,22]. Then, BOLD-MRI volumes are analyzed using an iterative algorithm for temporal decomposition [20]. BOLD-CVR is defined as the %BOLD signal change per mmHg CO_2_. Further technical details of BOLD-CVR acquisition and calculation can be reviewed in the supplementary file. Details on the MR acquisition parameters can be found in the Supplementary Material.

### 2.3. Masking of the Contrast-Enhancing Lesion (Volume-Of-Interest, VOI)

**Figure 1:**
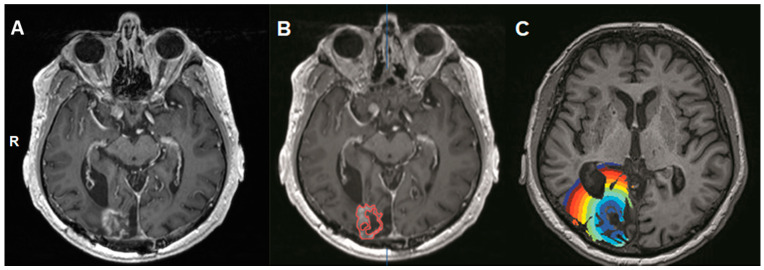
Illustration of the volumes-of-interest (VOIs). A contrast-enhanced axial T1-weighted morphological MRI scan of a patient with a right occipital contrast-enhancing lesion fitting the criteria for radiation necrosis (see methods). The three depicted images represent an axial orientation of T1-weighted volumes showing the right contrast-enhancing occipital lesion (**A**), the intralesional volume-of-interest drawn on the same scan (**B**), and the concentrically expanding 3 mm volumes-of-interest for the analysis of the perilesional tissue (**C**). For illustrative purposes, each volume-of-interest is depicted with a different color (R: right). This analysis was done for the radiation necrosis group and the group with newly diagnosed glioblastoma.

The contrast-enhancing lesion for both the radiation necrosis group as well as the glioblastoma group was manually drawn with iPlan software (BrainLab AG, Munich, Germany) on the contrast-enhanced high-resolution T1-weighted morphological MRI three-dimensional volume. The contrast-enhancing lesion was considered the intralesional mask in order to obtain an intralesional VOI (Figure 1).

Then, this intralesional mask was overlaid on the BOLD-CVR maps to obtain mean intralesional BOLD-CVR values for all patients. With an in-house written script using MATLAB2016, we performed an additional analysis of the perilesional tissue by creating concentrically expanding 3 mm VOIs starting from the contrast-enhancing lesion VOI outwards up to a maximum of 30 mm (see Figure 1C). These regions were automatically overlaid on the BOLD-CVR map to measure BOLD-CVR mean values, based on our previously published analysis concept [13,16]. Using predefined algorithms obtained from SPM 12 (Statistical Parameter Mapping Software, Welcome Department of Imaging Neuroscience, University College of London, London, UK), gray matter and white matter probability maps were derived. Using a combined map, a threshold of 0.9 was applied (e.g., the probability of it being either a gray matter or white matter voxel) for the region of interest to only include gray or white matter voxels. This allowed for a simple exclusion of voxels containing cerebrospinal fluid (CSF) or tissue outside of the brain.

### 2.4. Data Analysis

The Shapiro–Wilk test was used to assess the normal distribution of the data. A paired *t*-test was performed between mean values to assess for relevant differences in both intergroup and between VOIs (*p* < 0.05).

**Figure 2:**
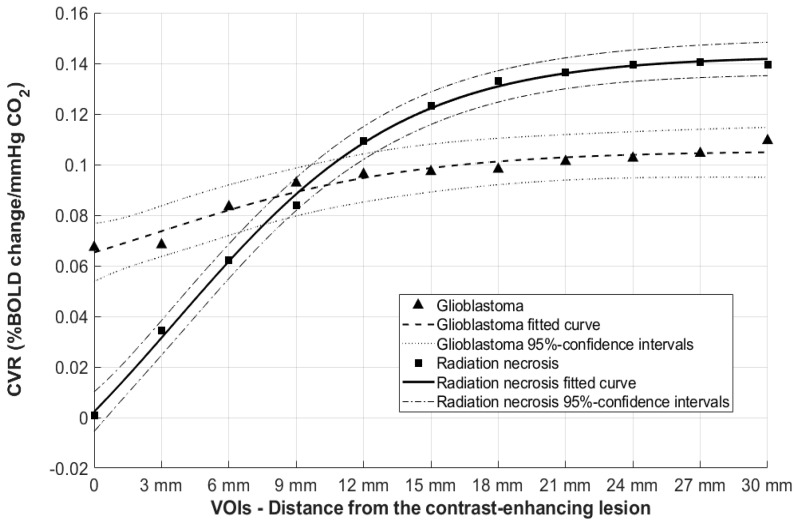
Scatter plot of mean CVR values and regression analysis for glioblastoma and radiation necrosis groups: blood oxygenation-level dependent fMRI cerebrovascular reactivity (BOLD-CVR) values were calculated as %BOLD signal/mmHgCO_2_. The *x*-axis represents the volumes-of-interest (VOIs) in which the BOLD-CVR values were measured. BOLD-CVR values are mean values of the two cohorts. The sigmoidal fit curves parameters are the lowest (a) and the highest (b) points of the curve, the curve midpoint (d), and the curve steepness (k). (GBM: glioblastomas, RN: radiation necrosis).

A sigmoidal fit *(y = a + (b − a) × 1/{1 + exp[−k × (x − d)]})* regression was applied to determine the goodness of fit, resulting in a model generating steepness of the curve (defined as k, see also Figure 2 and Supplemental Material), the sigmoidal mid-point (d), as well as the lowest (a), and highest (b) BOLD-CVR values of the sigmoidal fit on an individual subject basis. The CVR changing rate could be calculated as (b − a)/k. To assess the possible influence of outliers in the regression model, Cook’s distance was used. Receiver Operating Characteristic (ROC) and area under the curve (AUC) were calculated. The F1 score, indicating the harmonic mean between precision and the recall matrix, which describes the optimal cut-off point for an ROC curve, was determined. Diagnostic properties of our model, such as sensitivity, specificity, accuracy, and positive and negative predicting value, were determined. All calculations were performed with MATLAB2013 (The MathWorks, Inc., Natick, MA, USA; www.matworks.com, accessed on 6 February 2018) and SPSS Statistics 23 (IBM Corp. Armonk, NY, USA).

## 3. Results

Eight consecutive patients with primary or secondary brain tumors and multidisciplinary diagnosis of radiation necrosis (4 female) were enrolled, with a mean age of 55 years (36–77). Further relevant clinical details can be reviewed in Table 1. This patient cohort was compared to 14 subjects with newly diagnosed glioblastoma grade IV WHO (4 female), with a mean age of 67.2 (48–79). All glioblastoma patients of the reference cohort underwent surgical resection of their lesion, making a histopathological diagnosis confirmation possible.

### BOLD-CVR Findings for Patients with Radiation Necrosis and Newly Diagnosed Glioblastoma

For the radiation necrosis group, mean BOLD-CVR values in the contrast-enhancing lesion were markedly lower than for the contrast-enhancing lesion in the newly diagnosed glioblastoma group (0.001 ± 0.06 vs. 0.057 ± 0.05; *p* = 0.04).

The concentric ring analysis showed a marked BOLD-CVR improvement further away from the contrast-enhancing lesion, whereas for the glioblastoma group, the BOLD-CVR impairment showed almost no difference as compared to the primary VOI (i.e., the contrast-enhancing lesion). These data are illustrated in Figure 2 and Table 2.

**Figure 3:**
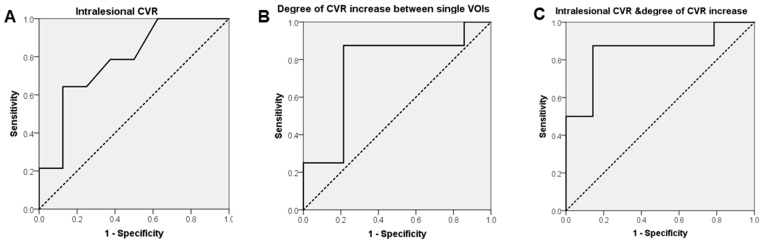
Receiver Operating Characteristic curve for BOLD-CVR in the contrast-enhancing lesion, CVR changing rate, and for the combination of the two parameters. (**A**) Area under the curve (AUC) of CVR in the contrast-enhancing part of the lesion (AUC: 0.78, 95% CI: 0.57–0.99); diagonal segments are produced by ties. (**B**) AUC of the CVR changing rate in the analyzed VOIs (AUC: 0.76, 95% CI: 0.53–0.99. (**C**) Intralesional CVR and CVR increase rate throughout the expanding VOIs were analyzed together by performing a binary logistic regression and obtaining probability estimates (AUC: 0.85, 95% CI: 0.65–1.00).

For the ROC curve analysis, the area under the curve showed a good ability of the intralesional BOLD-CVR in classifying the patients (Figure 3).

The sigmoidal fit model described well the mean CVR rates in the two groups (R^2^-radiation necrosis: 0.99; R^2^-glioblastomas: 0.99; see also Supplemental Material). All individual patient parameters (lowest CVR value, highest CVR value, curve mid-point, steepness of the curve) derived from the fit can be reviewed in the supplementary Table S1. Cook’s distance showed the presence of a single outlier, which did not influence the overall fit (see Figure S1 in Supplementary Material). The binary logistic regression combining the intralesional CVR values with the CVR changing rate from the contrast-enhancing areas toward the external VOIs allowed us to obtain a model based on probability estimates and showing a better discriminative capacity (AUC: 0.85, 95% CI: 0.65–1.00, see Figure 3). For this model, sensitivity and specificity were 0.50 (0.17–0.84) and 0.93 (0.66–1.00). Positive and negative predictive values were 0.77 (0.32–0.96) and 0.79 (0.65–0.88), respectively, and the accuracy was 0.79 (0.56–0.93). The calculated F1 score was 0.61.

## 4. Discussion

In this study, different intralesional and perilesional BOLD-CVR patterns were found for radiation necrosis contrast-enhancing lesions as compared to newly diagnosed glioblastoma contrast-enhancing lesions. We want to emphasize that the current dataset represents preliminary findings and that currently, no clinical merit can be derived from it. Our findings may be considered as a first step in describing patterns of radiation necrosis in patients with primary and secondary brain tumors. These distinct radiation necrosis BOLD-CVR patterns can open new research avenues to further validate this technique in precisely differentiating between radiation necrosis and glioblastoma recurrence.

### 4.1. BOLD-CVR and Vascular Pathophysiology in Glioblastoma and Radiation Necrosis

Both patients with radiation necrosis and glioblastoma showed a very distinct BOLD-CVR pattern within the contrast-enhancing lesion, but especially in the perilesional tissue, since both disease entities have different biological features. While radiation necrosis is thought to represent a focal tissue inflammatory response, glioblastoma is a diffuse infiltrative disease with an erratic perilesional growth pattern. This different biological behavior is coherently reflected in our BOLD-CVR findings. Here, the BOLD-CVR pattern for the radiation necrosis group improves dramatically in the immediate perilesional tissue, thereby indicating a more confined inflammatory response. Perilesional BOLD-CVR patterns for glioblastoma lesions do not show apparent improvement in the perilesional tissue, confirming previous findings by our group and others [12,15,16,17,23]. It is important to mention that these BOLD-CVR values are still a magnitude of an order lower than what is known to be normal reactivity in healthy subjects of similar age (mean whole-brain BOLD-CVR value for a healthy cohort = 0.23 ± 0.03) [7].

BOLD-CVR measures the brain vessels’ ability to modulate blood flow by changing their caliber after a vasoactive stimulus [21,24,25]. In glioblastomas, the vasodilatory capacity can be impaired due to neo-angiogenesis, which describes the formation of pathological vessels with blood–brain barrier (BBB) disruption and loss of regulative capacity [12,14,26,27,28,29].

Parallel to this, glioblastomas display a higher blood flow than the rest of the brain, related to hypermetabolism. Such high metabolic demand can cause peritumoral vessels to drop their perfusion pressure (i.e., dilate) in order to recruit sufficient blood flow.

Moreover, tumor cells are also found beyond the borders of a contrast-enhancing lesion without damaging the BBB, due to the infiltrative and aggressive behavior of such lesions [30,31]. Such cells may disrupt the perivascular organization due to the co-option of tumor cells accumulating around the existing vasculature, and this can result in impaired perilesional BOLD-CVR [32].

Instead, radiation necrosis is histologically characterized by coagulative and liquefactive necrosis involving predominantly the white matter, vascular hyalinization, fibrinoid deposition, and calcification [4] with lower blood flow [33]. These features result in nonresponding vascular tissue, which in turn can cause more impaired intralesional BOLD-CVR values. Since radiation necrosis is a focal disease, the perilesional tissue shows a quick normalization of BOLD-CVR. Indeed, our findings confirmed this hypothesis by showing lower intralesional CVR values in the radiation necrosis group (*p* = 0.04) and a more rapid CVR increase around the lesion. On the other hand, the glioblastoma group showed smaller circum-lesional variations without statistical difference between investigated VOIs (see also Figure 2 and Table 2). When combined, these two features (i.e., the difference in intralesional and perilesional BOLD-CVR patterns) allow for correct discrimination (AUC: 0.85, 95% CI: 0.65–1.00).

### 4.2. Current Advances in Follow-Up Management of Glioblastoma

In patients undergoing post-surgical radiotherapy for glioblastoma, the appearance of new contrast-enhancing lesions on clinical follow-up imaging poses a significant challenge. For instance, the diagnosis of radiation necrosis needs to be considered after a follow-up of 4 to 9 months with stable MR findings [2], but this strategy might be inappropriate for some patients with real tumor recurrence. Since therapies must be adapted according to the presence of tumor progression/recurrence versus treatment-related changes, obtaining a correct non-invasive diagnosis remains the ultimate goal. Many advanced functional MR imaging techniques have been studied in recent years. For instance, the potential of MR perfusion has been widely tested. Here, relative cerebral blood volume (rCBV) is measured in a region of interest selected by the operator inside the lesion after visual inspection of the scan, and the value is compared to that of a contralateral region of interest (ROI) [4]. In previous studies, a ratio of <0.6 between the two observed values has been proposed to identify radiation necrosis, and tumor recurrence was suspected for values >2.6 [34]. Nevertheless, drawbacks limit MRP’s reliability, such as its user-dependent nature, which exposes this technique to some biases in the selection of the ROIs35, the need for a contralateral “unaffected” hemisphere and standard acquisition methods are still lacking [35].

More recently, other imaging methods have been proposed. For instance, the use of magnetic resonance spectroscopy (MRS) has also shown some impressive results, and recent investigations have detected different ratios of metabolites in the tumors and radiation necrosis areas. Specifically, choline (Cho)/creatinine (Cr) and cho/N-acetyl-aspartate (NAA) were higher in recurrent tumors [33,36]. Here, a combination of a threshold of 1.17 for Cho/NAA and 1.11 for Cho/Cr allowed detecting tumor recurrence with a sensitivity of 83% and a specificity of 83% [37,38], and an integration of diffusion-weighted imaging and MRS was also proposed to further enhance the discrimination of pure radiation necrosis from lesions with mixed components (i.e., tumor and radiation necrosis) [39]. However, limitations in the spatial resolution are associated with this technique, especially for lesions under 1 cm of maximum diameter [4]. In addition, a long acquisition time exceeding 30 min may limit clinical implementation [5].

With regard to PET imaging, ^18^Fluoroethyl^18^Fluoroethyl-l-Tyrosine PET (FET-PET) radiotracer uptake was found to be significantly lower in patients with pseudo-progression than in glioblastomas with a sensitivity of 100% and a specificity of 96%, and also, time to peak (TTP) in tracer uptake was significantly lower in pseudoprogression [9] and is now considered an advanced method to identify tumor recurrence. Despite these promising results, later investigations have mentioned limitations, mainly because hypermetabolism can be associated with radionecrotic lesions, especially when subclinical seizure activity is present [40].

## 5. Limitations

The small cohort of patients with radiation necrosis (*n* = 8) and glioblastoma (*n* = 14) is a relevant limitation; however, it is suitable for a proof of concept study. Moreover, a definitive diagnosis of radiation necrosis is possible only with histological confirmation, which was not available here. However, the reliable inclusion criteria for radiation necrosis patients allow for a representative clinical diagnosis of radiation necrosis [2]. Additionally, patients enrolled in the radiation necrosis group differed on the disease for which radiotherapy was performed (i.e., primary brain tumors, meningiomas, or metastasis). This might have influenced the intrinsic BOLD-imaging patterns compared to glioblastomas in such patients. Even though no pathophysiological explanation justifies at present the possible occurrence of significantly different CVR responses among these radiation necrosis patients, this hypothesis must be taken into consideration and will need further investigations in the future. Even though absolute CVR values may vary significantly among radiation necrosis patients with different lesions being irradiated, possibly reflecting different influences of the tumor on the pathophysiology, similar CVR patterns were observed among subjects, with lower CVR values in the contrast-enhancing part, and more rapid increased perilesional compared to the reference group of newly diagnosed glioblastomas, supporting our hypothesis.

In patients with recurrent glioblastoma, the contrast-enhancing lesion most likely harbors both tumor tissue combined with radiation-induced changes. Therefore, our results need to be interpreted with caution and have no clinical merit based on this dataset. We selected newly diagnosed glioblastoma patients as a reference to be able to maximally exploit the different biological features between radiation necrosis and glioblastoma tissue. The goal of the study was to test the hypothesis of BOLD-CVR as a viable tool to characterize radiation necrosis: the potentials of BOLD-CVR in differentiating the hemodynamic features of suspected radiation necrosis versus tumor tissue to delineate a possible pattern should be studied in greater detail in future validation studies.

## 6. Conclusions

In this preliminary analysis, distinctive intralesional and perilesional BOLD-CVR patterns are found for radiation necrosis. The properties of BOLD-CVR as an adjunct tumor imaging parameter to identify radiation necrosis is promising but requires further evaluation.

## Figures and Tables

**Table 1:** Characteristics of the patient group with radiation necrosis. “Follow-up” refers to the time between completion of radiotherapy and the last follow-up scan, in which no progression of the disease was observed (Gy: Gray; GBM: Glioblastoma; WHO: World Health Organization; M/F: Male/Female).

Patient	Diagnosis	Sex/Age	Follow-Up (Months)	Radiotherapy Protocol
1	Atypical Meningioma (WHO grade II)	M/36	50	60 Gy—proton therapy
2	Anaplastic Oligodendroglioma (WHO grade III)	M/28	12	60 Gy
3	Meningotheliomatous meningioma (WHO grade I)	M/72	77	54 Gy
4	Anaplastic Oligodendroglioma (WHO grade III)	M/53	24	54 Gy
5	Brain metastasis (breast cancer)	F/56	11	radiosurgery (1 × 20 Gy)
6	Brain metastasis (breast cancer)	F/51	34	18 Gy interhemispheric + 20 Gy cerebellar
7	Glioblastoma (WHO grade IV)	F/77	10	40.5 Gy
8	Glioblastoma (WHO grade IV)	F/61	8	60 Gy

**Table 2:** Comparison of intralesional versus perilesional BOLD-CVR values.

Concentric VOI from the Contrast-Enhancing Lesion	Glioblastoma Mean CVR Value	Glioblastoma (*p*-Value)	Radiation Necrosis Mean CVR Value	Radiation Necrosis (*p*-Value)
VOI 1 (3 mm)	0.06	0.96	0.03	0.24
VOI 2 (6 mm)	0.07	0.50	0.06	0.04
VOI 3 (9 mm)	0.08	0.70	0.08	0.02
VOI 4 (12 mm)	0.09	0.89	0.11	0.01
VOI 5 (15 mm)	0.09	0.97	0.12	0.002
VOI 6 (18 mm)	0.10	0.97	0.13	<0.001
VOI 7 (21 mm)	0.10	0.89	0.14	<0.001
VOI 8 (24 mm)	0.10	0.95	0.14	<0.001
VOI 9 (27 mm)	0.10	0.93	0.14	<0.001
VOI 10 (30 mm)	0.11	0.83	0.14	<0.001

Relevant differences between BOLD-CVR perilesional value of individual VOIs as compared to the intralesional (i.e., contrast-enhancing) VOI. Significant differences were found only in the radiation necrosis group starting from 6 mm on, away of the contrast-enhancing lesion (*p* < 0.05 values are highlighted in bold digits) (VOI: volume of interest).

## Data Availability

Data is contained within the article or Supplementary Material.

## Supplementary Materials

The following are available online at https://www.mdpi.com/article/10.3390/cancers13081840/s1, Figure S1: Cook’s Distance showed only one significant outlier among the data (patient 11, see also the supplementary table), which however did not significantly alter the fit of the model, Table S1: The parameters of the sigmoid fits are presented for every patient and the mean of the CVR values in the two groups.
