# Supplementary material for: Distinct Cerebrovascular Reactivity Patterns for Brain Radiation Necrosis
Source: Cancers (Basel). 2021 Apr 13;13(8):1840. doi: 10.3390/cancers13081840 (PMC8069508; doi:10.3390/cancers13081840)
Supplement: Supplementary file 1 [file cancers-13-01840-s001.pdf]

Supplemental Material: Distinct Cerebrovascular Reactivity Patterns for Brain Radiation Necrosis

Giovanni Muscas, Christiaan Hendrik Bas van Niftrik, Martina Sebök, Alessandro Della Puppa, Katharina Seystahl, Nicolaus Andratschke, Michelle Brown, Michael Weller [3,4], Luca Regli, Marco Piccirelli and Jorn Fierstra

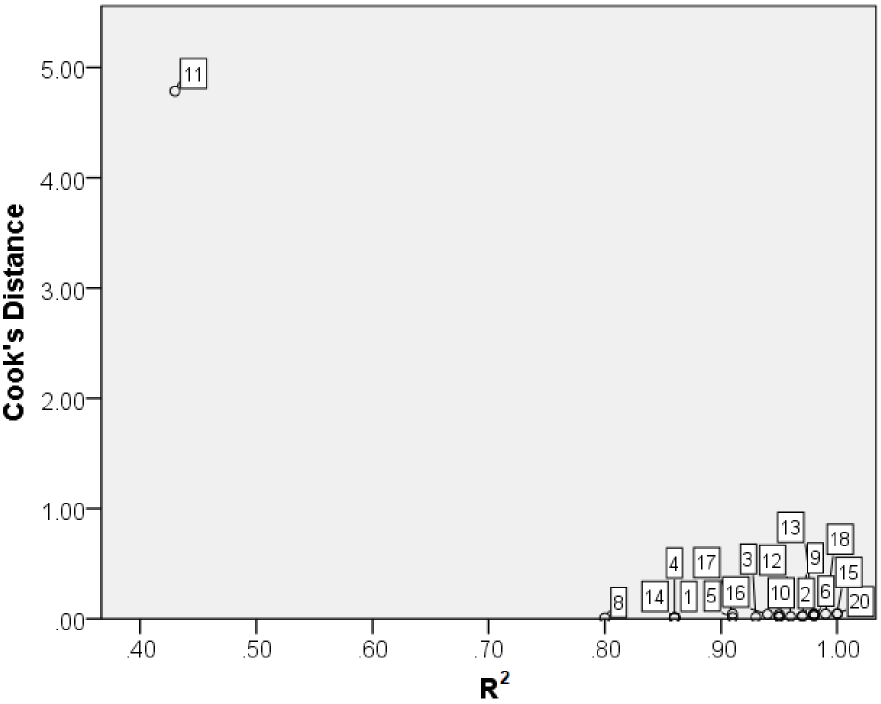

Figure S1. Cook's Distance showed only one significant outlier among the data (patient 11, see also the supplementary table), which however did not significantly alter the fit of the model.

Table S1. The parameters of the sigmoid fits are presented for every patient and the mean of the CVR values in the two groups (a: lowest CVR value; b: highest CVR value; d: curve midpoint; k: steepness of the curve; R^2: coefficient of determination [R-squared]; (b-a)/k: expresses the CVR changing rate from the contrast-enhancing lesion towards the outer VOIs).

	a	b	d	k	R^2	(b-a)/k
1. Glioblastoma 1	0.05 (±0.004)	0.08 (±0.03)	25 (±12)	0.29 (±0.6)	0.86	0.08
2. Glioblastoma 2	0.01 (±0.008)	0.07 (±0.005)	11 (±1)	0.72 (±0.6)	0.97	0.08
3. Glioblastoma 3	0.04 (±0.01)	0.14 (±0.05)	24 (±3)	0.45 (±0.5)	0.93	0.22
4. Glioblastoma 4	0.01 (±0.03)	0.05 (±0.005)	3 (±3)	0.7 (±1.3)	0.86	0.22
5. Glioblastoma 5	0.04 (±0.05)	0.10 (±0.006)	3 (±3)	0.72 (±1.3)	0.91	0.09
6. Glioblastoma 6	0.11 (±0.01)	0.19 (±0.005)	6 (±1)	0.94 (±0.7)	0.98	0.10
7. Glioblastoma 7	0.04 (±0.009)	0.11 (±0.02)	20 (±3)	0.35 (±0.3)	0.95	0.18
8. Glioblastoma 8	0.08 (±0.04)	0.15 (±0.01)	24 (±4)	−0.75 (±1.9)	0.80	−0.09
9. Glioblastoma 9	0.01 (±0.1)	0.27 (±0.04)	10 (±5)	0.19(±0.2)	0.97	1.34
10. Glioblastoma 10	0.02 (±0.01)	0.06 (±0.01)	12 (±2)	0.75(±0.9)	0.95	0.06
11. Glioblastoma 11	−0.04 (±0.28)	0.16 (±0.02)	14 (±9)	0.76 (±4.05)	0.43	−0.04
12. Glioblastoma 12	0.02 (±0.02)	0.20 (±0.02)	4 (±12)	0.21 (±0.2)	0.96	0.87
13. Glioblastoma 13	-0.04 (±0.03)	0.15 (±0.01)	1 (±18)	0.15 (±0.15)	0.98	1.25
14. Glioblastoma 14	0.01 (±0.01)	0.06 (±0.06)	24 (±9)	0.3 (±0.5)	0.86	0.18
Mean	0.01 (±0.1)	0.1 (±0.005)	0.09 (±17)	0.12 (±0.1)	0.99	0.76
15. Radiation necrosis 1	−0.02 (±0.7)	0.18 (±0.01)	7 (±3)	0.25 (±0.08)	1.00	0.81

16. Radiation necrosis 2	0.09 (±0.02)	0.13 (±0.01)	5 (±1.6)	0.76 (±0.08)	0.94	0.04
17. Radiation necrosis 3	−0.14 (± 0.4)	0.09 (±0.03)	15 (±12.1)	0.29 (±0.4)	0.91	0.80
18. Radiation necrosis 4	0.02 (±0.05)	0.19 (±0.01)	10 (±2.9)	0.25 (±0.1)	0.99	0.69
19. Radiation necrosis 5	−0.1 (±0.5)	0.14 (±0.08)	37 (±31.7)	0.12 (±0.2)	0.95	2.05
20. Radiation necrosis 6	0 (±0.01)	0.12 (±0.01)	15 (±1.1)	0.18 (±0.04)	1.00	0.70
21. Radiation necrosis 7	0.15 (±0.1)	−0.1 (±0.1)	20 (±6.6)	−0.12 (±0.1)	0.98	2.06
22. Radiation necrosis 8	0.02 (±0.05)	0.22 (±0.2)	8 (±1.9)	0.55 (±0.5)	0.98	0.37
Mean	−0.07 (±0.05)	0.14 (±0.004)	4 (±3)	0.19 (±0.05)	0.99	0.58

MR acquisition parameters:

Studies were acquired on a 3-tesla Skyra VD13 (Siemens, Erlangen, Germany) using a 32-channel head coil on a sagittal image with voxel size: 3 × 3 × 3 mm3, slice gap 0.3 mm. A high-resolution 3D T1-weighted anatomical image was acquired with the same orientation as the BOLD fMRI scan for co-registration and overlay purposes (voxel size: 0.8 × 0.8 × 1.0 mm3). Whole-brain BOLD volumes were collected with an axial 7.20 min 2D EPI (echo planar imaging) BOLD sequence with voxel size 3 × 3 × 3 mm3, acquisition of matrix 64 × 64, 35 slices with ascending interleaved acquisition, slice gap 0.3 mm, GRAPPA (generalized autocalibrating partially parallel acquisitions) factor 2 with 32 reference lines, adaptive coil combination, auto coil selection, TR 2000 msec, TE 30 msec, flip angle 85°, bandwidth 2368 Hz/Px, 220 volumes, and field of view 192 × 192 mm. For co-registration of the functional sequence, skull stripping, and overlay purposes, an anatomical T1-weighted MPRAGE (magnetization prepared rapid acquisition) sequence (voxel size 0.5 × 0.5 × 0.9 mm; field of view read 240 mm; slice thickness 0.90 mm; TR 1900.0 msec; TE 2.60 msec; filter: prescan normalize, flip angle 9 ; base resolution 256; phase resolution 100%; interpolation to 512 × 512; and PAT [parallel acquisition techniques] mode GRAPPA) from the clinical protocol was used. The field of view from the BOLD image acquisition was copied to the T1-weighted image for better early realignment of both images.

The pseudo-square wave consisted of a hypercapnic plateau of 10 mmHg CO2 from the subject's resting CO2 baseline (100 s) for 80 s followed by a return to the baseline for 300 s. Raw BOLD fMRI images were preprocessed using Statistical Parameter Mapping software (SPM 12, Wellcome Trust Centre for Neuroimaging, Institute of Neurology, University College London; http://www.fil.ion.ucl.ac.uk/spm/, accessed on 06-02-2018). Before realignment, slice timing correction was applied to correct for the interleaved acquisition. The T1-weighted image was then linearly registered to the mean BOLD volume for optimal multimodal alignment. Automated segmentation of the T1-weighted image yielded grey and white matter, cerebrospinal fluid, skull and skin probability maps. Spatial smoothing of BOLD-CVR was performed using a 6x6x6mm3 full-width half-maximum Gaussian Kernel.
